# Effect of taping as treatment to reduce breast cancer lymphedema: literature review

**DOI:** 10.1590/1677-5449.007217

**Published:** 2018

**Authors:** Jaya Paula Thomaz, Tamires dos Santos Maximo Dias, Laura Ferreira de Rezende

**Affiliations:** 1 Centro Universitário das Faculdades Associadas de Ensino – FAE, Departamento de Fisioterapia, São João da Boa Vista, SP, Brasil.

**Keywords:** lymphatic system, athletic tape, lymphedema

## Abstract

Lymphedema is the most common complication during the postoperative period after surgery for breast cancer and can have a direct impact on daily activities. The objective of this study was to review the use of taping as an alternative/complementary treatment to reduce lymphedema. A literature review was conducted of scientific articles indexed on the PubMed, LILACS, MEDLINE, and PEDro databases and Google Scholar, and nine articles were selected. It was found that taping is a complementary therapy for reducing lymphedema, which may be used as an alternative treatment method, but cannot substitute multilayer compression therapy.

## INTRODUCTION

 Lymphedema secondary to breast cancer is one of the complications that can emerge after lymph node resection surgery, because of structural changes or changes to lymphatic function. It may be present in 12 to 30% of women after surgical treatment for breast cancer and its consequences can include difficulty performing daily activities, inability to engage in normal activities, and emotional problems such as unhappiness, frustration, constant reminders that recovery from the disease has not yet occurred, and lowered self-esteem, causing deterioration in both physical and psychological aspects of quality of life. 1 ^-^ 5 Its chronic nature requires treatment with multidisciplinary support, directed at achieving improvement in clinical status and quality of life. 

 The current gold standard treatment is complex physiotherapy (CP), a combination of care for the skin, manual lymph drainage _(MLD), compressive elastic and inelastic bandaging, and myolymphokinetic exercises_. 1 ^,^ 3 ^,^ 4

 Taping is a technique that was developed by the chiropractor Kenzo Kase in the 1970s and is the basis of the Kinesio Taping® method that was initially used by orthopedists and therapists to provide muscle support without restricting movements. The method gained popularity in the sporting community in Japan, after it was used by the volleyball team at the Olympics. Towards the end of the 1990s, it began to spread to Europe, Asia, and America, and both its use and research into its effects are growing steadily. 3 ^,^ 4 ^,^ 6

 Taping is a technique that consists of applying neurofunctional elastic bandages for orthopedic dysfunctions, but which has been adopted in clinical practice for dysfunctions in other systems, including the lymphatic system. The tape used is made from 100% cotton, water resistant, hypoallergenic, thermoadhesive, and stretchable longitudinally. It has similar weight and thickness to skin, and it has an elasticity of up to 140%, the same as skin. The adhesive layer absorbs body heat and so it can only be activated once, when it reaches body temperature, after the tape is rubbed. It can be left on the skin for 3 to 5 days, with an interval of 24 hours between one application and the next. 4 ^,^ 6

 Taping is being used in clinical practice as a complementary technique for treatment of lymphedema, hard or static edemas, fibrous scarring, and for edema that is difficult to access in areas of the face, sternum, and thorax. However, it is contraindicated in cases of tissue fragility, skin infections, tumoral lesions, history of allergy to the product, diabetes mellitus, renal failure, and uncontrolled systemic arterial hypertension. 4 ^,^ 6

 The combination of the elasticity of taping with skin stretching provokes elevation of the skin, producing increased space between the dermis and epidermis, by creating small folds known as convolutions. The space that is released produces reductions in pressure, enabling lymph flowing at high pressure in the interstitial space to displace into the area of lower pressure. Raising the skin, in combination with body movements, makes the connective tissue structurally more flexible, thereby forming a path to guide the lymph through connective tissue. This process causes the valves to open in the small initial lymph vessels, directing the flow of lymph, which can drain for 24 hours and be absorbed by the skin, which is where 80% of the lymph vessels are found. 2 ^-^ 4 ^,^ 6

 The elasticity of the athletic tape thus relieves compression of painful mechanical receptors, alleviating pain, increasing lymph movement, facilitating body movements, increasing space in the skin, and softening tissues. 3

 The objective of this study is to conduct a literature review of scientific articles that describe use of taping as an alternative treatment method for reducing lymphedema secondary to breast cancer. 

## METHODS

 Articles were selected for analysis from the scientific databases, PubMed, LILACS, MEDLINE and PEDro, and Google Scholar. The keywords cancer and/or neoplasm were combined with Kinesio Taping®, lymphtaping, taping, athletic tape, lymphatic system, lymph, and lymphedema, with no date limits. 

**Figure 1:**
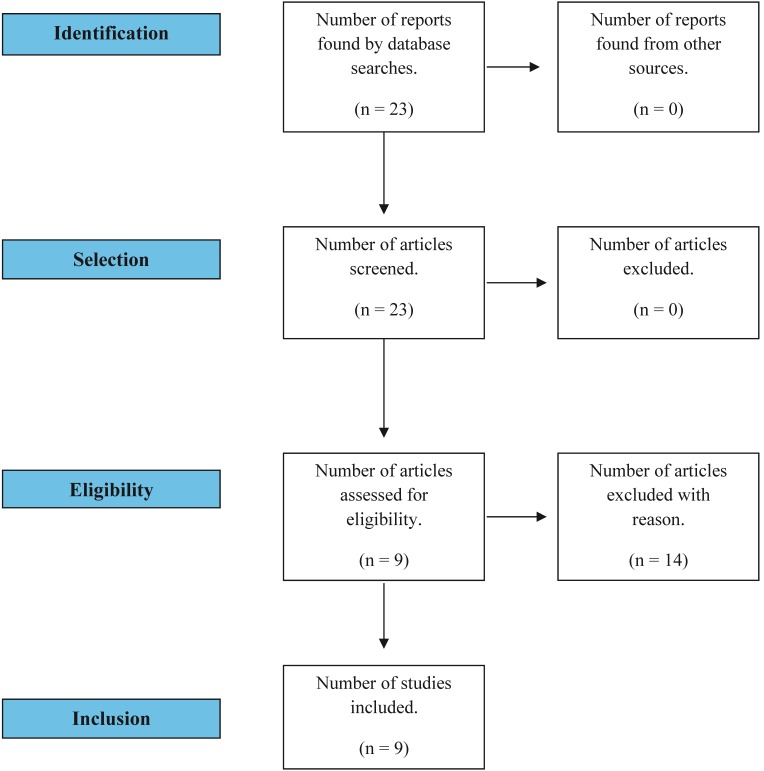
Flow diagram illustrating search and selection of studies.

 The inclusion criteria for studies were the treatment approach using the Kinesio Taping® */*lymphtaping/taping technique as a treatment for lymphedema secondary to breast cancer and comparative studies of this and other forms of treatment ( Figure 1 ). Articles dealing with patients with types of cancer other than breast cancer or with metastatic cancer were excluded. 

 Articles with a variety of evidence levels were included in order to encompass all of the scientific articles published on the subject. 

## RESULTS

 A total of nine original articles were identified in the databases that covered use of taping as method or in association with the gold standard treatment for reduction of lymphedema secondary to breast cancer. Table 1 describes the study designs and the methods of tape application. 

**Table 1:** Methods of taping applications.

**Authors**	**Methods**	**Taping application**
Finnerty et al. 7	10 people with lymphedema	Application of taping to direct lymph to a region with less congested lymph nodes.
Smykla et al. 8	Randomized clinical trial with 65 women with lymphedema with > 20% difference between limbs, divided into three groups: taping; without taping; CP	Taping is applied to the forearm with tension of 5-15% and left on the skin for 3 days.
Pekyavas et al. 9	Randomized clinical trial with 45 patients with grade II and III lymphedema, divided into three groups: CP; taping combined with CP; taping only	Kinesio Taping® method for lymphatic correction.
Taradaj et al. 10	Randomized clinical trial, with 70 patients with lymphedema, divided into three groups: taping and MLD; MLD and CPI; CP and CPI	Taping of the whole arm and forearm region
Martins et al. 11	24 patients with lymphedema	Taping applied on the skin of the anterior and posterior trunk, with the objective of stimulating formation of axillo-axillary anastomoses, and from proximal to distal on the upper limb, in the region opposite to normal physiology of lymph flow.
Pop et al. 12	Case study	Taping applied to hand, arm, and trunk, with longitudinal pressure of 30-40% in the longitudinal direction.
Do et al. 13	Randomized clinical trial with 44 patients with lymphedema: with spiral taping and traditional taping.	Application of spiral taping: four strips of taping along the length of the arm, at 45-degree angles, with 10% pressure, and directed to facilitate lymphatic drainage. Kinesio Taping® method for lymphatic correction.
Malicka et al. 14	Randomized clinical trial with 28 patients with grade I lymphedema, divided into two groups with two intervention subsets.	Taping applied with 1 cm width at the base, divided into four tails. The tension used was 15%, in the base-to-tail direction. 1st subset: taping applied over the lymphedema with individual tails on arm, forearm, and trunk 2nd subset: taping applied over the lymphedema with individual tails on the arm and forearm
Taradaj et al. 15	Case study	*Taping* applied to the anterolateral surface of the upper limb. The anchor was placed on the anterior surface of the hand, without tension. The tails were applied on the anterior, medial, and posterior surfaces of the arm and forearm, and the anterior thorax, with tension of 5-15%.

Table 2 , summarizes the results observed. 

**Table 2:** Results of application of the taping technique.

**Authors**	**Results**
Finnerty et al. 7	Satisfactory lymphedema reduction results in 70% of patients.
Smykla et al. 8	Use of taping was not effective for reduction of lymphedema, suggesting that it could be used as a complementary technique to CP. The 24.45% reduction in edema was not significant in relation to the other groups.
Pekyavas et al. 9	Use of taping in conjunction with standard treatment produced more satisfactory results (p = 0.008), and increased the effect of treatment. There was no significant difference between the groups in terms of symptoms related to lymphedema or satisfaction with treatment.
Taradaj et al. 10	Taping may be a good option for patients who are resistant to or have contraindications against CP. A 22.45% reduction in limb volume (p = 0.000118) and 24.13% reduction of edema (p = 0.00041). Reduction not significant in relation to other groups.
Martins et al. 11	Increased upper limb functionality was observed with taping (p < 0.001), but there was no difference in limb volume (p = 0.638).
Pop et al. 12	A 55% reduction in the volume of the limb with edema was observed, with the spiral technique producing better results. The result was significant in relation to the conventional taping subset (55% vs. 27% - p < 0.001).
Do et al. 13	There were improvements in quality of life and functional capacity, in addition to a 79.5% reduction in edema volume.
Malicka et al. 14	Taping is effective for lymphedema in the initial stages (p = 0.0009) and could be a safe alternative to CP when it is contraindicated.
Taradaj et al. 15	Taping was effective for reduction of lymphedema (reduction of 627 cm^3^ ) in 3 weeks. Edema reduction can be accelerated using taping.

## DISCUSSION

 There is little scientific evidence on use of the taping technique as an alternative treatment for lymphedema during the postoperative period of breast cancer. There is just one meta-analysis of the efficacy and safety of using taping in patients with lymphedema secondary to malignant neoplasm, which showed that taping appears to be superior to CP in terms of symptoms, but that patients who were treated with CP had better quality of life. 16 There are just five randomized clinical trials on the subject, with significant differences in relation to the method of application of the taping technique. The studies do exhibit consensus that the tape should be applied to the limb with edema. 

 Taping appears to be most effective for lymphedema in the initial stages and as a complement to CP. It does not appear to be more comfortable than compressive bandaging and its use also requires greater care. It is a safe and well-tolerated method for use with cancer patients. Taping should be considered as a complementary/alternative treatment, since it increases the space between the skin and muscle, and promotes increased blood and lymph flow, 2 ^,^ 4 ^-^ 6 increasing absorption of interstitial liquid, and lymph flow. 4 ^-^ 6 It could be a technique to be combined with the gold-standard method for treatment of lymphedema secondary to breast cancer. 

 Some disadvantages observed were that taping could make patients self-conscious, because it is visible, and body hair can interfere with tape adhesion. 6 Martins et al. 11 demonstrated a low incidence of skin problems and good patient tolerance. Neither skin damage or hyperthermia are common, but redness can occur frequently. 16

 Taping enables the lymphatic vessels to open, because the skin is lifted, facilitating lymph flow through improved microcirculation, in addition to guiding the lymph to the desired destination. Individual patient differences and daily activities should be taken into account. Experience with the technique is necessary to achieve the best results, taking into consideration the quality of the tape used, patient tolerance, and the method of application. 

## CONCLUSIONS

 Taping is an alternative complementary technique for reduction and maintenance of lymphedema secondary to breast cancer, which can be used as an adjuvant, but cannot substitute CP. Further studies of the technique are still needed, with descriptive explanations of the methods of application, and comparisons of the different types of applications with taping. 
