# Supplementary material for: Effect of taping as treatment to reduce breast cancer lymphedema: literature review
Source: J Vasc Bras. 2018 Apr-Jun;17(2):136–40. doi: 10.1590/1677-5449.007217 (PMC6205707; doi:10.1590/1677-5449.007217)
Supplement: Translation

# Translation: Efeito do uso do *taping* na redução do volume do linfedema secundário ao câncer de mama: revisão da literatura

**Authors:** Jaya Paula Thomaz, Tamires dos Santos Maximo Dias, Laura Ferreira de Rezende

**Affiliations:** 1 Centro Universitário das Faculdades Associadas de Ensino – FAE, Departamento de Fisioterapia, São João da Boa Vista, SP, Brasil.

**Keywords:** sistema linfático, fita atlética, linfedema

## Abstract

O linfedema é a complicação mais frequente no pós-operatório de câncer de mama, podendo afetar diretamente as atividades diárias. O objetivo desse estudo foi verificar o uso do *taping* como forma alternativa/auxiliar de tratamento na redução do linfedema. Foi realizada uma revisão da literatura de artigos científicos indexados nas bases de dados PubMed, LILACS, MEDLINE, PEDro e Google Acadêmico, onde foram selecionados nove artigos científicos. Verificou-se que o uso do *taping* é uma técnica complementar na redução do linfedema, podendo ser uma forma alternativa de tratamento para a redução deste, apesar de não ser capaz de substituir a terapia compressiva multicamadas.

## INTRODUÇÃO

 O linfedema secundário ao câncer de mama é uma das complicações que pode aparecer após cirurgia de ressecção de linfonodos, devido a alterações estruturais ou de função linfática. Pode estar presente de 12 a 30% das mulheres submetidas ao tratamento cirúrgico do câncer de mama, e pode ter como consequência dificuldades nas atividades diárias, privação das atividades usuais e alterações emocionais, como tristeza, frustração, lembrança constante da não recuperação da doença e piora da autoimagem, progredindo, assim, para a piora na qualidade de vida tanto em aspectos físicos quanto psicológicos 1 ^-^ 5 . Devido à sua cronicidade, é necessário que o tratamento tenha suporte multiprofissional, com o objetivo de promover evolução do quadro clínico e da qualidade de vida. 

 Atualmente o tratamento padrão-ouro é a terapia física complexa (TFC), uma combinação de cuidados com a pele, drenagem linfática manual _(DLM), enfaixamento compressivo elástico e inelástico e exercícios_ miolinfocinéticos 1 ^,^ 3 ^,^ 4 . 

 O *taping* é uma técnica desenvolvida pelo quiroprático Kenzo Kase como base do método Kinesio Taping®, que surgiu por volta da década de 1970, inicialmente aplicado por ortopedistas e terapeutas com o objetivo de promover suporte muscular sem gerar restrição do movimento. O ganho da popularidade do método surgiu na comunidade de esportes do Japão, após o uso deste pela equipe de vôlei em uma edição dos Jogos Olímpicos. A partir do final da década de 1990, o método se espalhou para Europa, Ásia e América, sendo cada vez mais utilizado e estudado 3 ^,^ 4 ^,^ 6 . 

 O *taping* é uma técnica aplicada por bandagens elásticas neurofuncionais para disfunções ortopédicas, mas que tem sido utilizado na prática clínica para disfunções de outros sistemas, inclusive o sistema linfático. A fita de *taping* é um material composto de 100% de algodão, resistente a água, hipoalérgico, termoadesivo e com alongamento no sentido longitudinal. É similar em espessura e peso à pele, com propriedade elástica de até 140%, correspondente à mesma da pele. A camada adesiva absorve o calor do corpo, portanto é ativada uma única vez, após atingir a temperatura corporal, depois que a fita foi esfregada. Pode permanecer na pele de 3 a 5 dias, com descanso de 24 horas entre uma aplicação e outra 4 ^,^ 6 . 

 Na prática clínica, está sendo utilizado como técnica complementar no tratamento de linfedemas, edemas duros ou estáticos, cicatrizes, fibroses e para edemas que são de difícil acesso em regiões de face, esterno e tórax. Por outro lado, é contraindicado em caso de fragilidade tecidual, infecções cutâneas, lesões tumorais, história de alergia ao produto, diabetes melito, insuficiência renal e hipertensão arterial sistêmica não controlada 4 ^,^ 6 . 

 A associação da elasticidade do *taping* com o estiramento cutâneo provoca a elevação da pele, promovendo um aumento entre os espaços da derme e da epiderme, mecanismo esse chamado de circunvolução. O espaço que foi liberado garante que haja uma redução na pressão e, com isso, permite que o fluxo linfático com pressão elevada no interstício se desloque para área de menor pressão. Com a elevação da pele e a combinação com os movimentos realizados pelo corpo, o tecido conjuntivo se torna mais flexível estruturalmente, formando assim um percurso de orientação para a linfa no tecido conjuntivo. Esse processo gera a abertura das válvulas dos pequenos vasos linfáticos iniciais e a linfa é direcionada, podendo ser drenada durante 24 horas e absorvida pela pele, onde se encontram 80% dos vasos linfáticos 2 ^-^ 4 ^,^ 6 . 

 Sendo assim, a elasticidade da fita *taping* promoveria a descompressão de receptores mecânicos e dolorosos, levando à diminuição da dor, ao aumento do movimento linfático, auxiliando nos movimentos corporais, ao aumento nos espaços da pele e ao amolecimento do tecido 3 . 

 O presente trabalho tem como objetivo realizar uma revisão da literatura de artigos científicos que relacionem o uso de *taping* como forma de tratamento alternativo para a redução do linfedema secundário ao câncer de mama. 

## MÉTODOS

 Foram analisados artigos na base de dados científicos, PubMed, LILACS, MEDLINE, PEDro e Google Acadêmico. Foram cruzadas as palavras-chaves câncer e/ou neoplasia com Kinesio Taping®, *linfotaping*, *taping*, *athletic tape,* sistema linfático, linfa e linfedema, sem restrição de período de tempo. 

 Os critérios de inclusão para os estudos encontrados foram a abordagem terapêutica do uso da técnica de Kinesio Taping®*/linfotaping*/ *taping* como forma de tratamento do linfedema secundário ao câncer de mama e estudos comparativos desta e de outras formas de tratamento ( Figura 1 ). Artigos com pacientes com outros tipos de câncer que não o de mama ou com câncer metastático foram excluídos. 

**Figura 1:** Fluxograma de busca e seleção de estudo.

 Foram incluídos artigos com variados níveis de evidências, para englobar todos os artigos científicos publicados sobre o assunto. 

## RESULTADOS

 Foram encontrados nove artigos originais nas bases de dados consultadas que abordavam a utilização do *taping* como meio ou associado ao tratamento padrão-ouro para a redução de linfedema secundário ao câncer de mama. A Tabela 1 descreve o desenho do estudo e a forma de aplicação do *taping* . 

**Tabela 1:** Forma de aplicação do *taping*.

**Autor**	**Método**	**Aplicação do *taping***
Finnerty et al. 7	10 pessoas com linfedema	Aplicação do *taping*, direcionando a linfa para a região de linfonodos menos congestionados.
Smykla et al. 8	Ensaio clínico randomizado com 65 mulheres com linfedema com diferença > 20% entre os membros, divididas em três grupos: *taping* ; sem *taping*; TFC	A aplicação foi realizada no antebraço com tensão de 5-15% do *taping* e deixada na pele por 3 dias.
Pekyavas et al. 9	Ensaio clínico randomizado com 45 pacientes com linfedemas graus II e III, divididos em três grupos: TFC; *taping* associado ao TFC; apenas o *taping*	Método Kinesio Taping® de correção linfática.
Taradaj et al. 10	Ensaio clínico randomizado, com 70 pacientes com linfedemas, divididos em três grupos: *taping* e DLM; DLM e CPI; TFC e CPI	*Taping* em toda região de braço e antebraço
Martins et al. 11	24 pacientes com linfedema	*Taping* foi aplicado na pele do tronco, anterior e posteriormente, com o objetivo de estimular a formação de anastomoses axiloaxilares, e de proximal para distal no membro superior, na região oposta à fisiologia normal do fluxo linfático.
Pop et al. 12	Estudo de caso	*Taping* aplicado na mão, braço e tronco, com pressão longitudinal de 30-40% sentido longitudinal.
Do et al. 13	Ensaio clínico randomizado com 44 pacientes com linfedema: com *taping* espiralado e taping tradicional.	Aplicação do *taping* espiralado: quatro tiras de *taping* ao longo do comprimento do braço, anguladas a 45 graus, com 10% de pressão e direcionadas para facilitar a drenagem linfática. Técnica de correção linfática Kinesio Taping®.
Malicka et al. 14	Ensaio clínico randomizado com 28 pacientes com linfedema grau I, divididos em dois grupos com dois subgrupos para a intervenção.	*Taping* aplicado com 1 cm de largura na base, dividido em quatro caudas. A tensão utilizada foi de 15%, no sentido da base para a cauda. 1º subgrupo: *taping* aplicado sobre o linfedema com caudas individuais sobre o braço, o antebraço e o tronco 2º subgrupo: *taping* aplicado sobre o linfedema com caudas individuais sobre o braço e antebraço
Taradaj et al. 15	Estudo de caso	*Taping* aplicado na região anterolateral do membro superior. A âncora foi colocada na região anterior da mão, sem tensão. As caudas foram aplicadas nas regiões anteriores, mediais e posteriores no braço e antebraço, e na região anterior do tórax com tensão de 5-15%.

 Na Tabela 2 , são discutidos os resultados encontrados. 

**Tabela 2:** Resultados da aplicação da técnica *taping* .

**Autor**	**Resultados**
Finnerty et al. 7	Resultados satisfatórios na redução do linfedema em 70% das pacientes.
Smykla et al. 8	A utilização do *taping* foi ineficaz para a redução do linfedema, sugerindo que possa ser uma técnica complementar ao TFC. Redução de 24,45% do edema, não significativa em relação ao demais grupos.
Pekyavas et al. 9	O uso do *taping* em conjunto com o tratamento padrão apresentam resultados mais satisfatórios (p = 0,008), além de potencializar o efeito do tratamento. Não houve diferença significativa entre os grupos em relação aos sintomas relacionados ao linfedema e satisfação com o tratamento.
Taradaj et al. 10	*Taping* pode ser uma boa opção para pacientes com resistência ou contraindicação para o uso da TFC. Redução de 22,45% do volume do membro (p = 0,000118) e 24,13% do edema (p = 0,00041). Redução não significativa em relação ao demais grupos.
Martins et al. 11	Aumento da funcionalidade do membro superior foi observada com o uso do *taping* (p < 0,001), mas nenhuma alteração no volume do membro (p = 0,638).
Pop et al. 12	Foi observado redução de 55% volume do membro edemaciado, com melhores resultados na técnica espiralda. Resultado significativo em relação ao grupo *taping* convencional (55% x 27% - p < 0,001).
Do et al. 13	Houve melhora na qualidade de vida e na capacidade funcional, além de redução de 79,5% do volume do edema
Malicka et al. 14	*Taping* é eficaz para linfedemas em estágios iniciais (p = 0,0009), podendo ser uma alternativa segura para a TFC quando existe contraindicação ao seu uso.
Taradaj et al. 15	*Taping* foi eficaz para a redução do linfedema (redução de 627 cm^3^) em 3 semanas. A redução do edema pode ser acelerada com o uso do *taping*.

## DISCUSSÃO

 A técnica *taping* é uma forma alternativa com pouca evidência científica para o uso no tratamento de linfedema no pós-operatório de câncer de mama. Existe apenas uma metanálise sobre a eficácia e segurança do uso do *taping* em pacientes com linfedema secundário a neoplasia maligna, a qual demonstrou que o uso do *taping* parece ser superior ao uso da TFC em relação aos sintomas, mas os pacientes que realizaram TFC têm melhor qualidade de vida 16 . Existem apenas cinco ensaios clínicos randomizados sobre o assunto, com significativa controvérsia em relação à forma de aplicação da técnica *taping.* Nesses estudos há consenso que a fita *taping* seja aplicada sobre o membro edemaciado. 

 O *taping* parece ser mais eficaz para linfedemas em estágios iniciais e como complemento à TFC. Não parece ser mais confortável que o enfaixamento compressivo, e ainda precisa ser utilizado com maior cautela. É um método seguro e tolerável a ser utilizado em pacientes oncológicos. Como tratamento complementar/ alternativo, o *taping* deve ser considerado, já que aumenta o espaço entre a pele e o músculo, e promove o aumento do fluxo sanguíneo e linfático 2 ^,^ 4 ^-^ 6 , aumentando a absorção do líquido intersticial e fluxo linfático 4 ^-^ 6 . Pode ser uma técnica a ser somada ao método padrão-ouro para o tratamento do linfedema secundário ao câncer de mama. 

 Algumas desvantagens encontradas foram que o *taping* pode ser constrangedor aos pacientes, por ser visível, e a presença de pelo pode interferir na aderência da fita 6 . Martins et al. 11 demonstraram baixa incidência de alterações de pele e boa tolerância das pacientes. Não são frequentes o aparecimento de lesões cutâneas ou hipertermia da pele, mas vermelhidão pode ser frequente 16 . 

 O *taping* permite que as vias linfáticas se abram, em decorrência do levantamento da pele, favorecendo o fluxo linfático através da melhora da microcirculação, além de direcionar a linfa até o local desejado. Deve-se levar em consideração a individualidade de cada paciente e as atividades do cotidiano. É necessário conhecer a técnica para que se possam conseguir melhores resultados, levando em conta a qualidade da fita, a aceitação do paciente e a forma de aplicação. 

## CONCLUSÃO

 O *taping* é uma técnica alternativa e complementar na redução e manutenção do linfedema secundário ao câncer de mama, podendo ser utilizada como forma auxiliar, mas sem capacidade para substituir a TFC. Ainda são necessários mais estudos sobre a técnica, com explicações descritivas das formas de aplicações, e que comparem os diferentes tipos de aplicações com o *taping*.
